# Overexpression of the recently identified oncogene *REDD1* correlates with tumor progression and is an independent unfavorable prognostic factor for ovarian carcinoma

**DOI:** 10.1186/s13000-018-0754-4

**Published:** 2018-11-14

**Authors:** Bin Chang, Jiao Meng, Huimin Zhu, Xiang Du, Lili Sun, Lei Wang, Shugang Li, Gong Yang

**Affiliations:** 1 Department of Pathology, Fudan University Shanghai Cancer Center, Shanghai, 20032 China; 2 Cancer Institute, Fudan University Shanghai Cancer Center, Shanghai, 20032 China; 3 Department of Oncology, Shanghai Medical College, Fudan University, Shanghai, 20032 China; 4 Department of Pathology, Shihezi University School of Medicine, Shihezi, 832003 Xinjiang China; 5 Department of Preventive Medicine, Shihezi University School of Medicine, Shihezi, 832003 Xinjiang China; 6 Central Laboratory, the Fifth People’s Hospital of Shanghai, Fudan University, Shanghai, 200240 China

**Keywords:** REDD1, Ovarian cancer, Prognosis, Invasion, Migration

## Abstract

**Background:**

Regulated in development and DNA damage response (*REDD1*), a gene responding to hypoxia or multiple DNA damage events, was recently implicated in cancer development and progression. Previously, in vivo and in vitro experiments indicated that *REDD1* functions as an oncogene in ovarian cancer cells. However, the role of *REDD1* in cancer cell migration and invasion and in clinical significance of prognostic values is not examined in detail.

**Methods:**

We detected the *REDD1* protein expression by immunohistochemistry in 18 normal ovarian surface epithelium or fallopian tube epithelium specimens, 24 ovarian borderline tumors, and 229 ovarian cancers. Fisher’s exact test, logistic regression analysis, the Kaplan–Meier method, and the log-rank test were used to evaluate the association of REDD1 with clinical factors, overall survival and disease-free survival. The prognostic predictive value of REDD1 for ovarian cancer patients was evaluated using multivariate Cox proportional hazard regression models. REDD1 expression in HEY, HEY A8, SKOV3, SKOV3 ip1, OVCA429, OVCA433 and A2780 human ovarian epithelial cancer cell lines was detected by western blotting. The role of REDD1 in cell invasion and migration was assessed by transwell migration and invasion assays using SKOV3, A2780, HEY, HEYA8, and SKOV3-REDD1 with parental A2780-REDD1 HEY-REDD1i and HEY A8-REDD1i.

**Results:**

High expression of REDD1 was observed in 35.4% of primary ovarian carcinoma samples. Overexpression of cytoplasmic REDD1 in ovarian cancer was significantly associated with serous carcinoma (*P* < 0.001), late-stage disease (*P* < 0.001), ascites (*P* < 0.001), and partial or non-response to chemotherapy (*P* < 0.001). High cytoplasmic expression of REDD1 was correlated with poorer overall survival (*P* < 0.001) and disease-free survival (*P* < 0.001). The multivariate Cox proportional hazards regression analysis indicated that patients with high cytoplasmic REDD1 expression had a high risk of death (*P* < 0.001) and high risk of an event (i.e., recurrence, progression, or death) (*P* < 0.001). REDD1 was first reported as an independent prognostic factor in ovarian cancer patients. In addition, REDD1 overexpression enhanced ovarian cancer cell migration and invasion.

**Conclusion:**

REDD1 is an independent unfavorable prognostic factor in ovarian carcinoma and may promote ovarian cancer metastasis.

**Electronic supplementary material:**

The online version of this article (10.1186/s13000-018-0754-4) contains supplementary material, which is available to authorized users.

## Background

REDD1 (regulated in development and DNA damage response 1, also known as RTP801/Dig1/DDIT4) was first identified in 2002 [1, 2]. It is a stress related protein induced by hypoxia and multiple DNA damage stimuli and is expressed broadly in many human tissues [1]. The gene is located at human chromosome 10q24.33 and is homologous to two *Drosophila melanogaster* genes of unknown function, *Scylla* and *Charybde,* which are designated as Hox targets in the National Institutes of Health genetic sequence database GenBank [1]. As a potent repressor of the mechanistic target of rapamycin in complex 1 (mTORC1), REDD1 regulates cell growth, tumorigenesis, cell aging, and autophagy [3–6].

Previously, we demonstrated that REDD1 overexpression promoted cell proliferation, colony formation and decreased apoptosis in human ovarian epithelial cells. REDD1 overexpression also resulted in tumor formation from nontumorigenic immortal ovarian surface epithelial cells. Our data indicated that REDD1 behaved as an oncogene in ovarian cancer pathogenesis [7]. However, the expression and clinical significance of REDD1 in ovarian cancer has not been examined in detail, and the correlation between REDD1 expression in different locations (cytoplasm or nuclear) and clinical pathological factors remains unknown. The prognostic value of REDD1 expression in ovarian cancer by multivariate analysis remains unclear.

The purpose of this study was to evaluate the association between REDD1 expression in different locations and clinical pathological factors (including age, pathology diagnosis, tumor grade, disease stage, ascites, serum CA125 level, clinical response to chemotherapy) as well as overall survival(OS)and disease-free survival (DFS) for ovarian cancer patients. To achieve this goal, we retrospectively analyzed 229 primary ovarian carcinomas with clinical pathological factors and generated a tissue microarray with archived tissue specimens used for REDD1 expression examination by immunohistochemical staining. The results were correlated with clinical outcome, and the effect of REDD1 on ovarian cancer cell migration and invasion was also tested.

## Methods

### Patients and clinicopathologic data

Based on the availability of representative tumor samples and follow-up information, the paraffin-embedded tissues of 229 primary ovarian cancers and 24 borderline tumors were collected at the Fudan University Shanghai Cancer Center between January 1, 1996 and December 31, 2013. Eighteen normal ovarian or fallopian tube tissues from patients who received an oophorectomy because of nonovarian tumor diseases were used as normal controls (Table 1). The relevant clinical data of 229 primary ovarian cancers are described in detail in Table 2. Follow-up information was updated through December 2016 by reviewing medical records and performing telephone follow-ups. The use of these clinical materials for research purposes was approved by the Institutional Research Ethics Committee. Histopathological diagnoses, and tumor grading of paraffin-embedded tissues were reassessed by two experienced gynecology pathologists (BC and LW) based on the World Health Organization classification of female reproductive organ tumors (4th edition, 2014) [8]. Serous carcinomas were grade as low grade and high grade, endometrioid and mucinous carcinomas were grade as I, II and III, and all clear cell carcinomas were graded as III. Disease staging was assigned according to TNM and the International Federation of Gynecology and Obstetrics staging system of the ovary, fallopian tube and primary peritoneal carcinoma [8]. Patients were defined as platinum-sensitive, partially sensitive, and resistant if the disease recurrence was ≥12, ≥6 and < 12, and < 6 months, respectively, after their last receipt platinum-based chemotherapy.

**Table 1:** REDD1 expression in different ovarian epithelial tissues

Pathological type	Total No.	Cytoplasmic REDD1 expression		*P*-value	Nuclear REDD1 expression		*P*-value
		Low expression No. (%)	High expression No. (%)		Negative No. (%)	Positive No. (%)	
Normal ovarian or fallopian epithelia	18	15(83.3)	3(16.7)	0.009	9(50)	9(50)	0.000
Borderline tumor	24	22(91.7)	2(8.3)		9(37.5)	15(62.5)	
Carcinoma	229	148(64.6)	81(35.4)		186(81.2)	43(18.8)

**Table 2:** Correlation between cytoplasmic REDD1 expression and clinicopathologic factors

Characteristic	REDD1 expression		Total No.	χ^2^	*P*-value
	Low expression No. (%)	High expression No. (%)			
Age					
20-	4(66.7)	2(33.3)	6		0.911^*^
30-	7(70.0)	3(30.0)	10		
40-	31(67.4)	15(32.6)	46		
50-	42(68.9)	19(31.1)	61		
60-	45(58.4)	32(41.6)	77		
70-	16(64.0)	9(36.0)	25		
80-	3(75.0)	1(25.0)	4		
Stage				20.283	< 0.001
Stage I	27(81.8)	6(18.2)	33		
Stage II	24(85.7)	4(14.3)	28		
Stage III	85(62.5)	51(37.5)	136		
Stage IV	12(37.5)	20(62.5)	32		
Histologic type					< 0.001^*^
Serous carcinoma	58(46.4)	67(53.6)	125		
Mucinous carcinoma	6(100.0)	0(0.0)	6		
Endometrioid carcinoma	30(96.8)	1(3.2)	31		
Clear-cell carcinoma	11(78.6)	3(21.4)	14		
Mixed-type carcinoma	43(81.1)	10(18.9)	53		
Ascites				18.699	< 0.001
Yes	75(54.7)	62(45.3)	137		
No	44(72.1)	17(27.9)	61		
Unknown	26(93.5)	2(6.5)	31		
Chemotherapy response					< 0.001^*^
Responders	119(74.8)	40(25.2)	159		
Partial responders	14(34.1)	27(65.9)	41		
Non responders	8(42.1)	11(57.9)	19		
NC/UR^a^	7(70.0)	3(30.0)	10		
CA125					
< 500	38(59.4)	26(40.6)	64		0.529^*^
≥ 500	38(58.5)	27(41.5)	65		
Unknown	72(72.0)	28(28.0)	100		

^*^Fisher’s exact test

^a^NC/UR: No chemotherapy or Unknown response

### Tissue microarray construction and immunohistochemical analysis

One core from a morphologically representative area of the paraffin-embedded block was taken from each patient to construct microarray blocks as previously described [9].

Then, 5 μm microarray slides were stained by immunohistochemistry according to the manufacturer’s protocol (Biocare Medical, Concord, CA, USA) [9]. In brief, after the initial deparaffinization/hydration, sections were microwaved for 15 min in 10 mM citrate buffer, pH 6.0, to unmask epitopes. Nonspecific binding was blocked with background sniper (Biocare Medical). Then, the slides were incubated overnight at 4 °C with a primary mouse monoclonal antibody against REDD1 (ab63059/ Anti-DDIT4 antibody, 1:100 dilution; Abcam pIc, Cambridge Science Park, UK) followed by incubation with a biotin-labeled secondary antibody (Universal Goat Link; Biocare Medical) for 15 min. Next, the slides were incubated with HRP (Biocare Medical) for 15 min. Tissues were stained for 5 min with 3′-diaminobenzidine (Biocare Medical), and the sections were counter-stained with hematoxylin, dehydrated, and mounted in glycerol-vinyl-alcohol (GVA mount, Zymed). Formalin-fixed, paraffin-embedded human lung carcinoma tissue that was robustly REDD1 positive was used as an internal positive control.

Immunohistochemical staining for REDD1 was analyzed by two pathologists (BC and LW). Cytoplasmic staining was scored according to the intensity and percentage of cancer cells. The intensity of cytoplasmic staining was quantified using a four-score grading system. Cores without REDD1-positive cells were given a score of 0. Samples with weakly REDD1-positive cells were scored 1, those with medium REDD1-positive cells were scored 2; and samples with strongly REDD1-positive cells were scored 3. The percentage of positively stained cancer cells was recorded. Cytoplasmic staining results were scored by multiplying the percentage of positive cells (P) by the intensity (I). For the statistical analysis, we split the cases into two groups: low expression (with scores≤1) and high expression (with scores> 1). Nuclear positive was defined as ≥5% tumor cell nucleus stained with REDD1 with any intensity (weak, moderate or strong).

### Cell culture and transfection

The human ovarian epithelial cancer cell lines HEY, HEYA8, SKOV3, SKOV3 ip1, OVCA429, OVCA433 and A2780 and the HEK293T were purchased from American Type Culture Collection (ATCC, USA). Cells were cultured in RPMI1640 medium (ovarian cancer cell lines) or Dulbecco’s Modified Eagle Medium (HEK293T) with 10% fetal bovine serum, 100 U/mL penicillin, and 100 μg/mL streptomycin at 37 °C in a humidified 5% CO2 atmosphere.

REDD1-overexpressing lentivirus was supplied by Hanyi (Shanghai, China). SKOV3 and A2780 were infected with the REDD1-overexpressing lentivirus (SKOV3-REDD1 and A2780-REDD1) or a corresponding mock lentivirus expressing EGFP (SKOV3- EGFP and A2780- EGFP). REDD1 short hairpin RNAs (shRNAs) were purchased from Genechem (Shanghai, China). HEY and HEYA8 were infected with the lentiviruses encoding REDD1 or control shRNA plasmids, and stable cell lines (HEY-REDD1i/HEY-shCon and HEY A8-REDD1i/HEY A8-shCon) were established. Transfection was performed with Fugene HD (Promega, CA) according to the manufacturer’s instructions. Plasmid was added to cells with 8 μg/mL Polybrene (Sigma-Aldrich, US) for 6–8 h, and then fresh culture medium was added. Forty-eight hours later, the culture was harvested, and targeted cells were infected and selected with puromycin.

### Western blot analysis

To analyze protein expression in cells, immunoblotting was performed according to standard methods [7]. The REDD1 primary antibody for immunoblotting (10638–1-AP) was obtained from Proteintech. Actin (A2228, Sigma Aldrich, US) was used as a loading control. Anti-rabbit (cs-7074) and anti-mouse (cs-7076) secondary antibodies bound to HRP were obtained from Cell Signaling Technology (Massachusetts, US). The protein bands were visualized with chemiluminescent reagents (Millipore, US).

### Transwell migration and invasion assay

Cell migration was assayed using transwell chambers (8 μm pore size BD, US). Briefly, 5 × 10^4^ cells in 150 μL serum-free RPMI1640 medium were seeded into the upper chamber. The chamber was placed into a 24-well plate, and the lower wells contained RPMI1640 medium with 20% FBS. After 24 h of incubation, the cells on the upper surface of the chamber were carefully swabbed with a cotton swab. The cells migrating through the chamber were fixed with methanol, stained with crystal violet and subsequently counted from 5 different areas in each well with an inverted microscope. The mean of triplicate assays for each experimental condition was used. Similar inserts coated with Matrigel were used to determine invasive potential in the invasion assay.

### Statistical analysis

Fisher’s exact test and logistic regression analysis were performed to evaluate the association of REDD1 with clinical factors. The Kaplan–Meier method was used to estimate the probability of overall survival and disease-free survival, and the log-rank test was used to compare the overall survival or disease-free survival between different comparison groups, such as patients with low or high REDD1 expression. Multivariate Cox proportional hazards regression models were fitted to determine the significant factors associated with overall survival and disease-free survival and to assess REDD1 association with overall survival or disease-free survival after adjusting for the effect of other clinical factors. The overall survival time (OS) was calculated as the time interval from the date of first biopsy to the date of death or last follow-up, whichever occurred first. Patients alive on the last follow-up date were censored. The disease-free survival time (DFS) was calculated as the time period from the date of first biopsy to the date of recurrence, the date of death, or the date of last follow-up, whichever occurred first. Patients alive on the last follow-up date without recurrence were censored. Results were considered statistically significant when *P* < 0.05. SAS 9.1 software (SAS Institute Inc., Cary, NC, USA) was used for statistical analysis.

## Results

### Patient characteristics

The median age of the 229 patients with ovarian carcinoma was 58.9 years (range 21.3–87.6 years). The median OS was 38.0 months (95% CI: 20.3–270.7 months). The median DFS was 17.8 months (95% CI: 0–213.4 months).

### REDD1 expression and localization

**Fig. 1:**
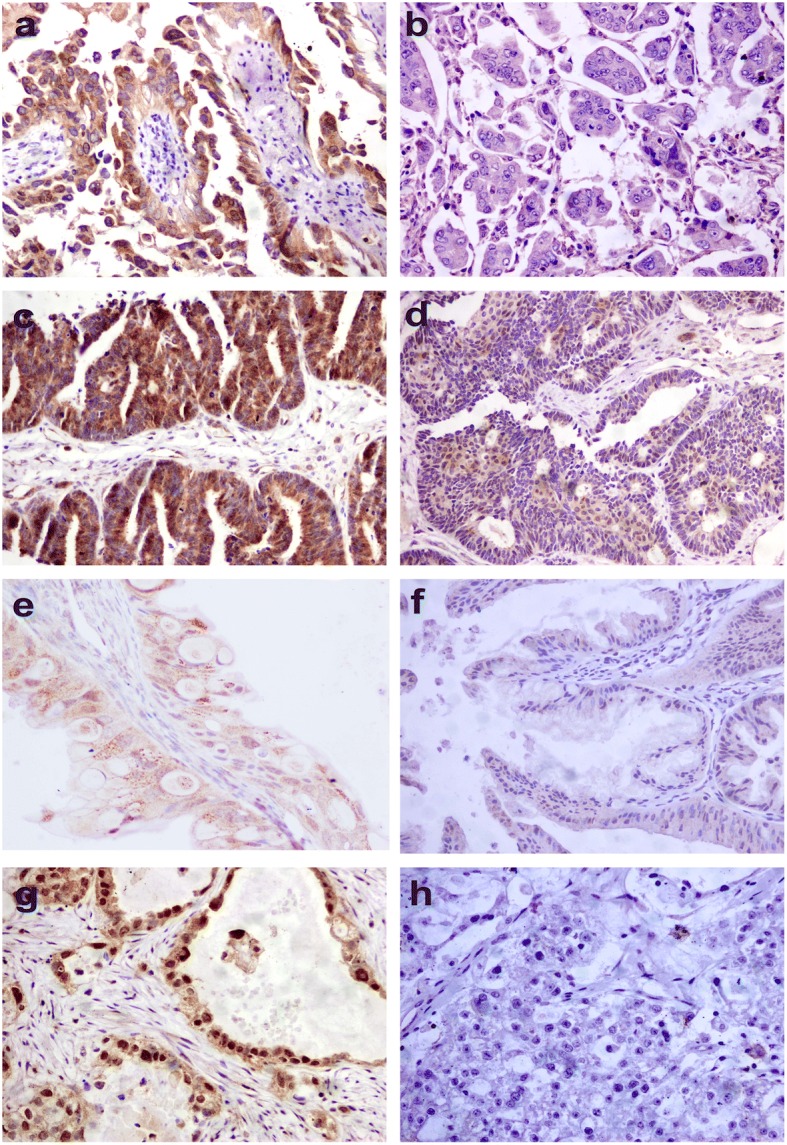
Immunoreactivity patterns of REDD1 in ovarian carcinomas. **a** REDD1-high expression in serous carcinoma. **b** Serous carcinoma cells show no REDD1 staining. **c** Diffuse and strong positive staining for REDD1 in endometrioid carcinoma. **d** Endometrioid carcinoma cells show very weak cytoplasmic REDD1 staining. **e** REDD1-positive staining in mucinous adenocarcinoma. **f** Mucinous adenocarcinoma do not exhibit REDD1 staining. **g** Positive nuclear staining for REDD1 in clear cell carcinoma. **h** Clear cell carcinoma cells do not show staining for REDD1 (original magnification × 200)

Cytoplasmic and nuclear REDD1 staining were observed with variable intensity (Additional file 1: Figure S1) in different proportions of tumor cells. The percentage of REDD1-positive cancer cells varied from 0~ 100% in our patient population (Fig. 1). In our series, only 18.8 (43/229) of ovarian carcinomas were positive for nuclear REDD1 in which two patterns including nuclear positive and cytoplasmic negative staining (3/43) and both cytoplasmic and nuclear positive staining (40/43). In order to clarify the clinical significance of REDD1 in different cellular localization, we analyzed the expression of REDD1 in cytoplasm and nucleus, respectively.

### REDD1 expression in normal ovarian surface epithelial tissue, borderline tumors and ovarian cancer

To investigate the association between REDD1 protein expression and ovarian cancer, we examined the tissue microarray by immunohistochemistry. In 18 normal or fallopian epithelia group, seven cases (38.9%) showed both cytoplasmic and nuclear positive, two cases (11.1%) showed both cytoplasmic and nuclear negative, seven cases (38.9%) showed only cytoplasmic positive, and two cases (11.1%) showed only nuclear positive. In 24 borderline tumors, nine cases (37.5%) showed both cytoplasmic and nuclear positive, four case (16.7%) showed both cytoplasmic and nuclear negative, five cases (20.8%) showed only cytoplasmic positive, and six (25%) showed only nuclear positive.

Cytoplasmic REDD1 expression was significantly higher in ovarian cancer specimens (35.4%, 81/229) than in normal ovarian surface epithelial tissue and fallopian tube tissue specimens (16.7%, 3/18) and borderline tumor tissue specimens (8.3%, 2/24) (*P* = 0.009, Table 1). Our previous data suggested that REDD1 was a key mediator required for the RAS-mediated transformation of ovarian epithelial cells. Now, our findings provide further support that REDD1 plays a significant role in epithelial ovarian cancer pathogenesis. Moreover, nuclear REDD1 positive was significantly lower in ovarian cancer specimens (18.8%, 43/229) than in normal ovarian surface epithelial tissue and fallopian tube tissue specimens (50%, 9/18) and borderline tumor tissue specimens (62.5%, 15/24) (*P* = 0.000, Table 1).

### Association between REDD1 expression and clinicopathologic ovarian carcinoma variables

The tumor microarray immunostaining results were organized according to patient clinicopathologic characteristics and shown in Tables 2 and 3. Cytoplasmic and nuclear staining in cancer cells were analyzed, and high cytoplasmic REDD1 expression was associated with serous carcinoma (*P* < 0.001), late-stage disease (*P* < 0.001) (the cut- off for early vs. late stage is I-II vs. III–IV), and ascites (*P* < 0.001). The correlation of REDD1 expression with response to primary therapy is shown in Table 2.

**Table 3:** Correlation between nuclear REDD1 expression and clinicopathologic factor

Characteristic	REDD1 expression		Total No.	χ^2^	*P*-value
	Negative	Positive			
	No. (%)	No. (%)			
Age					0.508^*^
20-	5(83.3)	1(16.7)	6		
30-	6(60.0)	4(40.0)	10		
40-	37(80.4)	9(19.6)	46		
50-	49(80.3)	12(19.7)	61		
60-	66(85.7)	11(14.3)	77		
70-	19(76.0)	6(24.0)	25		
80-	4(100.0)	0(0.0)	4		
Stage				3.4	0.334
Stage I	25(75.8)	8(24.2)	33		
Stage II	20(71.4)	8(28.6)	28		
Stage III	115(84.6)	21(15.4)	136		
Stage IV	26(81.3)	6(18.8)	32		
Histologic type					0.043^*^
Serous carcinoma	105(84.0)	20(16.0)	125		
Mucinous carcinoma	6(100.0)	0(0.0)	6		
Endometrioid carcinoma	24(77.4)	7(22.6)	31		
Clear cell carcinoma	7(50.0)	7(50.0)	14		
Mixed-type carcinoma	44(83.0)	9(17.0)	53		
Ascites				3.633	0.161
Yes	108(78.8)	29(21.2)	137		
No	49(80.3)	12(19.7)	61		
Unknown	29(93.5)	2(6.5)	31		
Chemotherapy response					0.801^*^
Completely Responders	130(81.8)	29(18.2)	159		
Partial responders	34(82.9)	7(17.1)	41		
Non responders	14(73.7)	5(26.3)	19		
NC/UR^a^	8(80.0)	2(20.0)	10		
CA125					0.750^*^
< 500	50 (78.1)	14 (21.9)	64		
≥ 500	54 (83.1)	11 (16.9)	65		
Unknown	82 (82.0)	18 (18.0)	100		

^*^Fisher’s exact test

^a^NC/UR: No chemotherapy or Unknown response

In total, 197 patients (86.0%) received postsurgical platinum-taxol-based treatment; either alone or in combination with other adjuvant drugs. Eight patients (3.5%) did not receive chemotherapy. In 21 patients (9.2%), platinum-taxol-based treatment was administered before surgical debulking surgery. In three patients (1.3%), the treatment protocol was unknown. High cytoplasmic REDD1 expression was associated with partial or non-response to chemotherapy (*P* < 0.001). No correlation was found between cytoplasmic REDD1 expression and patient age or serum CA125 levels (Table 2). High nuclear REDD1 expression was associated with clear cell carcinoma (*P* = 0.043). No correlation was observed between nuclear REDD1 expression and patient age, disease stage, ascites, or chemotherapy response (Table 3). We analyzed the correlation between the cytoplasmic expression of REDD1 and the grade of serous carcinoma and non-carcinoma separately because different grading systems were used for serous carcinoma and endometrioid carcinoma. No correlations were found between REDD1 expression and either serous or non-serous carcinoma grade (Additional file 2: Tables S1 and S2).

### High REDD1 expression correlates with poor patient survival

**Fig. 2:**
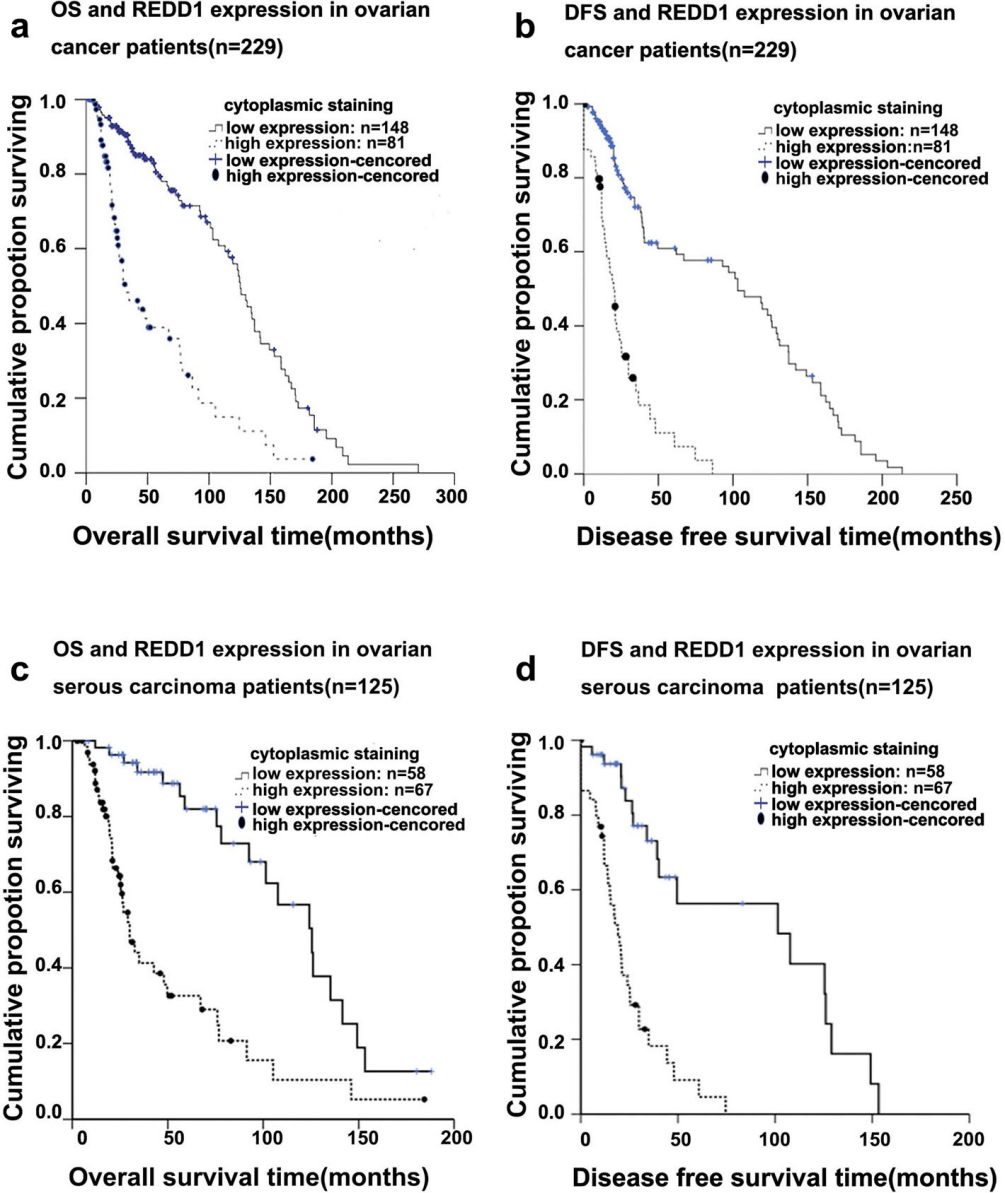
Kaplan–Meier survival curves ovarian carcinoma patients grouped by low and high REDD1 expression levels. **a** OS curves in all patients with ovarian cancer (*n* = 229). **b** DFS curves in all patients (*n* = 229). **c** OS curves in patients with ovarian serous carcinoma (*n* = 125). **d** DFS curves in patients with ovarian serous carcinoma (*n* = 125)

Overall survival and disease-free survival rates at 3, 5, and 10 years are shown in relation to cytoplasmic REDD1 expression in Tables 4 and 5. At the time of this report, 45 of the 229 analyzed patients were alive without clinical evidence of ovarian carcinoma or low malignant potential tumor, 39 were alive with ovarian carcinoma, 137 died of ovarian carcinoma, and 8 were alive with unknown ovarian carcinoma status. Cytoplasmic REDD1 expression was correlated with OS and DFS. Patients with high REDD1 expression had worse overall survival rates (*P* < 0.001) (Fig. 2a) and disease-free survival rates (*P* < 0.001) (Fig. 2b) than patients with low REDD1 expression. Then we analyzed the correlation between REDD1 expression (low vs. high) with OS and DFS in serous carcinoma group. Patients with high REDD1 expression had worse overall survival rate (*P* < 0.05) and disease free survival rate (*P* < 0.05) than patients with low REDD1 expression (Fig. 2c and d) in serous carcinomas group. Nuclear REDD1 expression (negative vs. positive) was not correlated with OS and DFS (Additional file 2: Tables S3 and S4).

**Table 4:** Cytoplasmic REDD1 expression and OS

REDD1 expression	No. of patients	Median survival months (95% CI)	Survival rate (95% CI)			χ2	*P*-value
			36-months	60-months	120-months		
Low expression	148	125.000(113.914,137.086)	0.76(0.671, 0.828)	0.71(0.007,0.976)	0.33(0.217,0.448)	40.115	< 0.001
High expression	81	32.700(16.536,48.864)	0.41(0.273,0.542)	0.24(0.119,0.384)	0.08(0.017,0.211)

**Table 5:** Cytoplasmic REDD1 expression and DFS

REDD1 expression	No. of patients	Median survival months (95% CI)	Survival rate (95% CI)			*χ* ^*2*^	*P*-value
			36-months	60-months	120-months		
Low expression	148	103.200(76.880,129.520)	0.76(0.671,0.828)	0.60(0.495,0.690)	0.27(0.161,0.391)	60.873	< 0.001
High expression	81	20.000(15.954,24.046)	0.41(0.292,0.524)	0	0

To investigate whether REDD1 is an independent prognostic factor, REDD1 expression and other clinical pathological parameters (age, stage, histologic type, ascites, serum CA125 level, chemotherapy response, and cytoplasmic REDD1 expression) were examined further in a multivariate analysis. The multivariate Cox proportional hazards regression analysis indicated that cytoplasmic REDD1 expression was significantly associated with OS (Table 6) and DFS (Table 7). Patients with high cytoplasmic REDD1 expression had a higher risk of death (*P* < 0.001) and a higher risk of an event (i.e., recurrence, progression, or death) (*P* < 0.001) than patients with low cytoplasmic REDD1 expression. High REDD1 expression was found to be an independent prognostic factor for poor OS and DFS.

**Table 6:** The multivariate Cox proportional- hazards regression for OS

							95.0% CI for Exp(B)	
	B	SE	Wald	df	*P*-value	Exp(B)	Lower	Upper
Age(years)								
< 40			12.931	2	0.002			
40–60	−1.99	0.594	11.217	1	0.001	0.137	0.043	0.438
≥ 60	−2.096	0.585	12.82	1	< 0.001	0.123	0.039	0.387
Histologic type								
Serous carcinoma			12.222	4	0.016			
Mucinous carcinoma	1.813	0.785	5.331	1	0.021	6.13	1.315	28.57
Endometrioid carcinoma	0.014	0.385	0.001	1	0.972	1.014	0.476	2.158
Clear-cell carcinoma	1.722	0.634	7.366	1	0.007	5.593	1.613	19.391
Mixed-type carcinoma	−0.121	0.415	0.085	1	0.771	0.886	0.393	1.997
Ascites								
No			6.469	2	0.039			
Yes	0.444	0.291	2.336	1	0.126	1.559	0.882	2.756
Unknown	−1.378	0.796	2.999	1	0.083	0.252	0.053	1.199
Stage (II-IVvsI)	1.554	.338	21.096	1	< 0.001	4.729	2.437	9.177
cytoplasmic REDD1 expression	1.201	0.318	14.23	1	< 0.001	3.325	1.781	6.206

Dependent variable: overall survival

Independent variables: age, stage, histologic type, ascites, serum CA125 level, chemotherapy response, cytoplasmic REDD1 expression

B:regression coefficient, SE:standard error, Wald:Wald chi square, Df: degree of freedom

**Table 7:** The multivariate Cox proportional- hazards regression for DFS

							95% CI for Exp(B)	
	B	SE	Wald	df	*P*-value	Exp(B)	Lower	Upper
Age (years)								
< 40			1.562	2.000	0.458	1.000		
40–60	0.488	0.394	1.536	1.000	0.215	1.629	0.753	3.524
≥ 60	0.040	0.229	0.031	1.000	0.860	1.041	0.665	1.629
Histologic type								
Serous carcinoma			16.706	3.000	0.001	1.000		
Mucinous carcinoma	0.380	0.450	0.713	1.000	0.399	1.462	0.605	3.533
Endometrioid carcinoma	1.953	0.587	11.065	1.000	0.001	7.053	2.231	22.297
Clear-cell carcinoma	1.192	0.700	2.900	1.000	0.089	3.292	0.835	12.972
Mixed-type carcinoma	0.656	0.483	1.844	1.000	0.174	1.926	0.748	4.961
Response to chemotherapy								
Completely Responders			17.056	3.000	0.001	1.000		
Partial responders	1.885	0.503	14.051	1.000	0.000	6.584	2.458	17.639
Non-responders	0.956	0.656	2.120	1.000	0.145	2.600	0.718	9.412
Unknown response	−0.839	0.672	1.559	1.000	0.212	0.432	0.116	1.613
Stage (II-IVvsI)	0.904	0.312	8.361	1.000	0.004	2.468	1.338	4.554
Cytoplasmic REDD1 expression	−1.479	0.295	25.102	1.000	< 0.001	0.228	0.128	0.407

Dependent variable: disease-free survival time

Independent variables: age, stage, histologic type, ascites, serum CA125 level, stage, chemotherapy response, and cytoplasmic REDD1 expression

B: regression coefficient, SE: standard error, Wald: Wald error, Wald: Wald chi square, Df: degree of freedom

Except for REDD1 expression, age, histological type, ascites,and stage were also confirmed to be independent prognostic factors for overall survival (Table 6, *P* = 0.002, *P* = 0.016, and *P* = 0.039, and *P*<0.001 respectively). Histologic type, response to chemotherapy, and stage were independent prognostic factors for disease-free survival (Table 7, *P* =0.001, *P*=0.001, and *P*=0.004 respectively).

### REDD1 enhances ovarian cancer cell migration and invasion

**Fig. 3:**
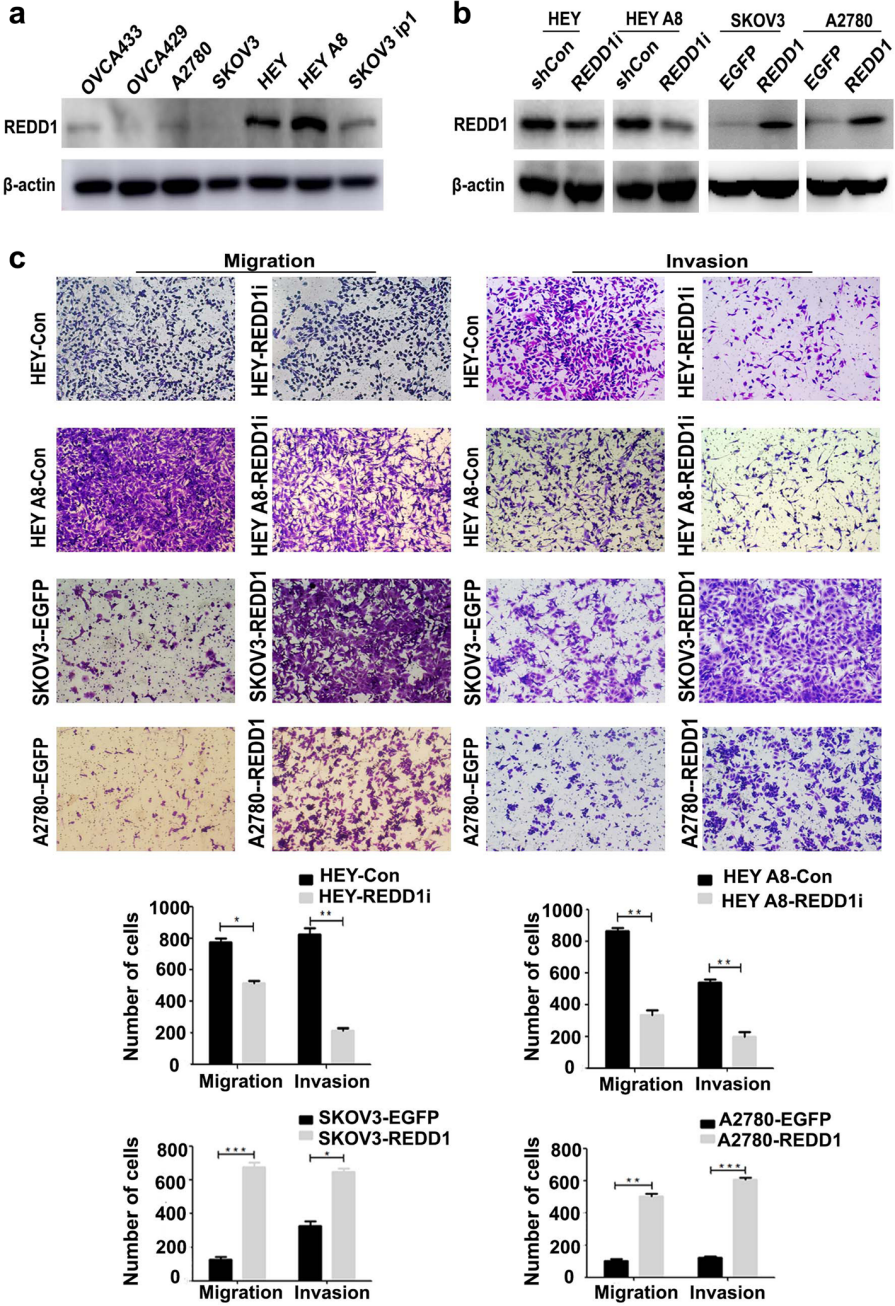
REDD1 enhance cell migration and invasion in ovarian cancer. **a** Western blotting detected REDD1 expression level in human ovarian epithelial cancer cell lines. **b** Construction of ovarian cancer cell lines with REDD1 overexpression or knockdown. **c** Transwell assays illustrate that REDD1 enhances ovarian cancer cell migration and invasion ability

We previously reported that REDD1 overexpression promoted cell proliferation and colony formation in human ovarian epithelial cells [7]. Because our data showed that high REDD1 expression correlated with poor ovarian cancer patient prognosis, we explored the effect of REDD1 on the migration and invasion of ovarian cancer cells. We detected the REDD1 expression level in seven human ovarian epithelial cancer cell lines (Fig. 3a). Low REDD1 expression was detected in OVCA433, OVCA429, A2780, and SKOV3 cell lines, whereas a high REDD1 was observed in HEY and HEYA8 cell lines. Using lentiviral infection, we transfected REDD1 cDNA into SKOV3 and A2780 and delivered shRNA targeting REDD1 into HEY and HEYA8. REDD1 was robustly overexpressed or silenced in cells treated with REDD1 cDNA or REDD1 shRNA (REDD1i), respectively, compared with control cells (Fig. 3b).

To investigate whether REDD1 activity is critical for cell migration and invasion, we performed transwell migration and invasion assays. As shown in Fig. 3c, more SKOV3-REDD1 and A2780-REDD1 cells migrated through the chamber membrane, and fewer HEY-REDD1i and HEY A8-REDD1i cells migrated than the corresponding control cells. Similar results were observed for the invasion assay, which support our claim that REDD1 may promote ovarian cancer metastasis through enhancing cell migration and invasion (*P* < 0.05).

## Discussion

In this study, we showed that REDD1 expression was significantly upregulated in ovarian cancer tissues compared with normal ovarian surface epithelial tissue and borderline tumors. Dr. Jia et al. reported that high REDD1 expression was correlated with a shorter DFS and OS in 100 ovarian cancer specimens by Kaplan Meier survival analysis; however, multivariate factor analysis was not performed to evaluate the value of REDD1 expression for ovarian cancer prognoses, and REDD1 expression in different locations (cytoplasm and nuclear) was not discretely correlated with the clinical pathologic factors and patient survival [10]. Here, we show that cytoplasmic REDD1 overexpression is an independent prognosis factor for ovarian cancer in 229 ovarian cancer specimens analyzed by multivariate Cox proportional hazards regression, indicating that REDD1 might be a prognostic biomarker for ovarian cancer patients. Furthermore, we show for the first time that REDD1 may promote ovarian cancer metastasis through upregulation of cell migration and invasion. Additionally, we analyzed the correlation of cytoplasmic and nuclear REDD1 expression separately with clinicopathologic variables. Our data showed that high cytoplasmic rather than nuclear expression of REDD1 was associated with serous carcinoma, late-stage disease, and partial or no chemotherapy response.

Michel Grégory et al. found that wild-type REDD1 localized to the cytoplasm and nucleus of cells before activation; plasma membrane translocation was triggered after activation [11]. We found nuclear REDD1 positive was significantly lower in ovarian cancer specimens than in normal ovarian surface epithelial tissue and fallopian tube tissue specimens and borderline tumor tissue specimens. Plasma membrane translocation might be a reason for this phenomenon. Ovarian clear cell carcinoma has a molecular pathogenesis distinctive from other histotypes of ovarian cancer, such as serous carcinoma. Here, we noted that high nuclear REDD1 expression was more common in clear cell carcinoma than in the other ovarian cancer histotypes. This result indicates that REDD1 may have different functions in different cellular locations. In the future, more cases should be included, and potential mechanisms of nuclear REDD1 expression in ovarian normal epithelia tissues, borderline tumor and carcinoma tissues should be further investigated.

REDD1 has been demonstrated to be a potent repressor of the protein kinase signaling pathway and is referred to as mTORC1 by the HIF1–REDD1–TSC1 axis [3, 12–17]. Abnormal expression of REDD1 occurs during the pathogenesis of multiple diseases. However, the biological significance in human cancer remains unclear. The roles of REDD1 in carcinogenesis seem paradoxical. Peter Horak [4] and Blanka Kucejova [18] showed that REDD1 suppressed tumorigenesis in breast cancer and sporadic clear cell renal cell carcinoma, respectively. However, Jin HO et al. [19] demonstrated that sustained REDD1 overexpression leads to mTORC1 inhibition and consequent Akt activation, which occurs to promote cell survival in lung cancer. Recently, REDD1 was reported to act as an oncogene in bladder urothelial carcinoma [20]. Depending on the cellular context, REDD1 has been shown to act as either an oncogene or tumor suppressor gene (Table 8).

**Table 8:** Research of REDD1 in different tumor types

First author	Journal (year)	Results	Function of REDD1	Tumortype
Horak P	Proc Natl Acad Sci U S A. (2010) [4]	• REDD1 inactivation induces ROS dysregulation and consequent HIF-1α induction that promotes tumorigenesis. • Loss of REDD1 induces a hypoxia-dependent increase in proliferation and anchorage-independent growth in vitro. • Breast carcinomas exhibit silencing of REDD1 expression compared with normal epithelia.	Suppresses tumorigenesis	breast cancer
Kucejova B	Mol Cancer Res. (2011) [18]	• REDD1 is highly expressed in VHL-deficient clear-cell renal cell carcinoma (ccRCC). • Mutations in REDD1 may contribute to ccRCC development.	possibly a tumor suppressor in sporadic ccRCC.	ccRCC
Jin HO	Cancer Lett. (2013) [19]	• Sustained overexpression of Redd1 leads to mTORC1 inhibition and to consequent Akt activation that is involved in cell survival. • Akt phosphorylation, which consequent to mTORC1 inhibition and sustained REDD1 overexpression, plays a role in cell survival and resistance to chemotherapeutic drugs.	/	lung cancer cells.
Zeng Q	Clin Cancer Res. (2018) [20]	• The significant increase of REDD1 expression is detected in bladder urothelial carcinoma(BUC) tissue. • REDD1 is an independent prognostic factor in BUC patients. • Silencing REDD1 expression in T24 and EJ cells decreased cell proliferation, increased apoptosis, and decreased autophagy. The ectopic expression of REDD1 in RT4 and BIU87 cells had the opposite effect. • Inhibited REDD1 expression sensitizes BUC tumor cells to paclitaxel in a subcutaneous transplant sarcoma model in vivo.	REDD1 is an oncogene. Antagonizing REDD1 could be a potential therapeutic strategy to sensitize BUC cells to paclitaxel	BUC

In this study, compared with borderline tumor and normal ovarian or fallopian tube epithelia, REDD1 expression was upregulated in ovarian carcinomas, whereas cytoplasmic REDD1 expression was significantly higher in serous carcinoma (53.6%) than in other histotypes (*p* < 0.001). Our results indicated that REDD1 might be a potential target for treatment in serous carcinomas. On the other hand, multivariate Cox proportional hazards regression analysis showed that cytoplasmic REDD1 expression was strongly associated with overall survival and disease-free survival (*P* < 0.001 and *P* < 0.001, respectively) and also adjusted by other variables (age, histologic type, response to chemotherapy, and stage). Our data show that cytoplasmic REDD1 expression was an independent predictor for OS and DFS, indicating that REDD1 has potential as a prognostic biomarker for ovarian cancer. Our results are similar to the report that REDD1 acts as an oncogene in bladder urothelial carcinoma and correlates with poor patient survival [20]. Man-ming Cao et al. used cDNA microarray analysis to show that REDD1 was upregulated in a cisplatin-resistant human ovarian carcinoma cell line [21]. Our data from 229 clinical specimens showed that high cytoplasmic REDD1 expression was associated with partial or non-response to chemotherapy (*P* < 0.001) in patients with ovarian carcinoma, suggesting that REDD1 plays an important role in ovarian cancer chemoresistance. More patient cases and experiments are required to validate these results, and the potential mechanism should be investigated further. Our results indicated that REDD1 might be a chemotherapy response predictor for ovarian cancer patients and is a potential therapeutic chemoresistance target in ovarian cancer patients.

## Conclusions

High REDD1 expression is associated with a poor prognosis for ovarian cancer patients, and might be a predictor of chemotherapy response for ovarian cancer patients. REDD1 may promote ovarian cancer metastasis through inducing cell migration and invasion.

## Additional files

Additional file 1: **Figure S1.** Immunoreactivity intensity of REDD1 in ovarian carcinomas. (a) REDD1 negative. (b) REDD1 weak staining. (c) REDD1 medium staining. (d) REDD1 strong staining. (e) Cytoplasmic REDD1 positive in cancer cells. (f) Both cytoplasmic and nuclear expression of REDD1 in cancer cells. (original magnification × 400). (JPG 24700 kb)

Additional file 2: **Table S1.** Correlation between cytoplasmic REDD1 expression and tumor grade in non-serous carcinomas. **Table S2**. Correlation between cytoplasmic REDD1 expression and tumor grade in serous carcinomas. **Table S3.** Nuclear REDD1 expression and OS. **Table S4**. Nuclear REDD1 expression and disease-free survival. (DOCX 26 kb)

## Abbreviations

DFS: Disease-free survival time
OS: Overall survival time
REDD1: Regulated in development and DNA damage response
