# Supplementary figures and images for: Overexpression of the recently identified oncogene REDD1 correlates with tumor progression and is an independent unfavorable prognostic factor for ovarian carcinoma
Source: Diagn Pathol. 2018 Nov 14;13:87. doi: 10.1186/s13000-018-0754-4 (PMC6236897; doi:10.1186/s13000-018-0754-4)

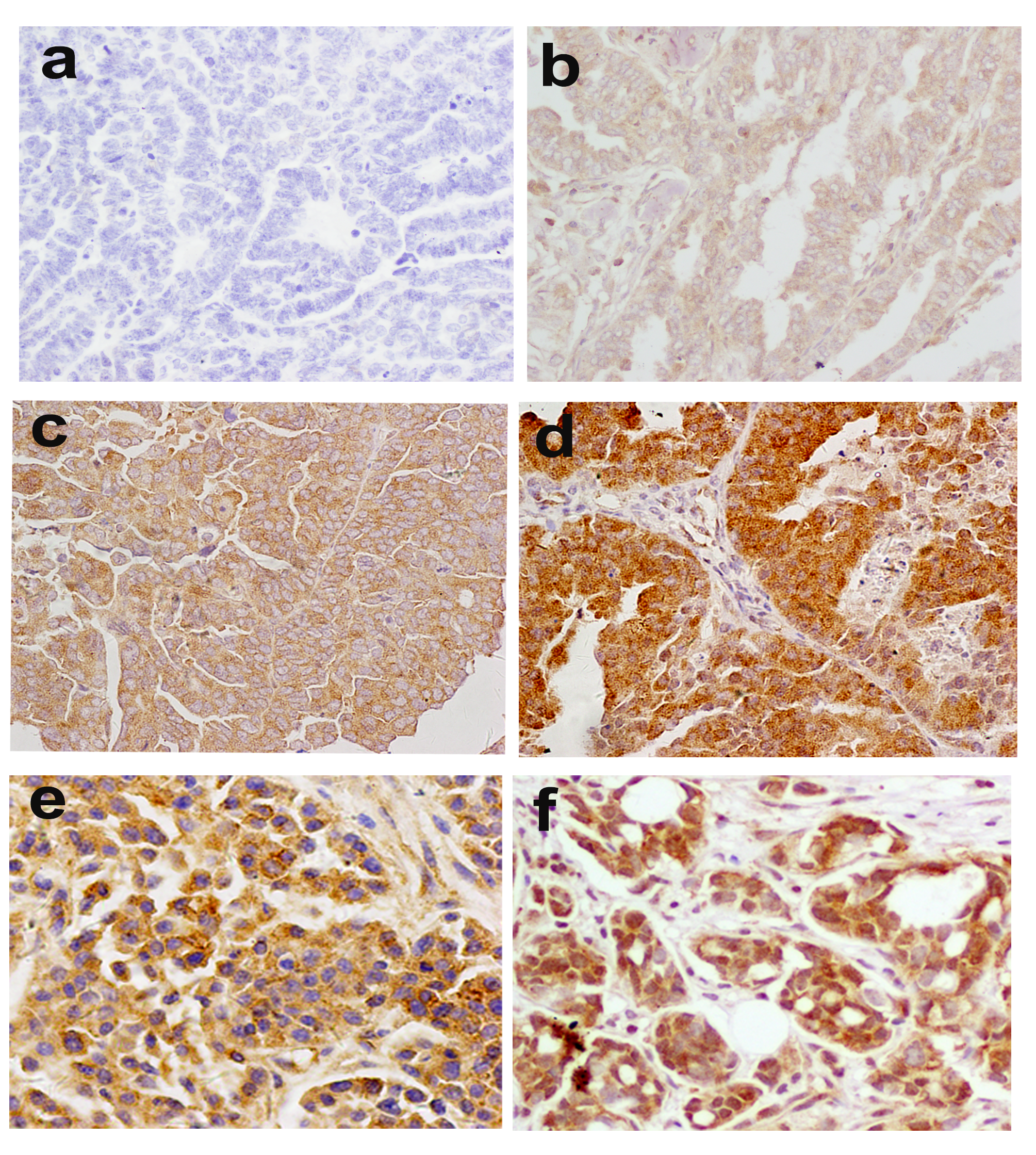

Supplement: Supplementary file 1 — Figure S1. Immunoreactivity intensity of REDD1 in ovarian carcinomas. (a) REDD1 negative. (b) REDD1 weak staining. (c) REDD1 medium staining. (d) REDD1 strong staining. (e) Cytoplasmic REDD1 positive in cancer cells. (f) Both cytoplasmic and nuclear expression of REDD1 in cancer cells. (original magnification × 400). (JPG 24700 kb) [file 13000_2018_754_MOESM1_ESM.jpg]
