# Supplementary material for: Overexpression of the recently identified oncogene REDD1 correlates with tumor progression and is an independent unfavorable prognostic factor for ovarian carcinoma
Source: Diagn Pathol. 2018 Nov 14;13:87. doi: 10.1186/s13000-018-0754-4 (PMC6236897; doi:10.1186/s13000-018-0754-4)
Supplement: Supplementary file 2 — Table S1. Correlation between cytoplasmic REDD1 expression and tumor grade in non-serous carcinomas. Table S2. Correlation between cytoplasmic REDD1 expression and tumor grade in serous carcinomas. Table S3. Nuclear REDD1 expression and OS. Table S4. Nuclear REDD1 expression and disease-free survival. (DOCX 26 kb) [file 13000_2018_754_MOESM2_ESM.docx]

**Table S1** Correlation between cytoplasmic REDD1 expression and tumor grade in

non-serous carcinomas

| Grade | REDD1 Expression | | Total No. | *P*-value |
| --- | --- | --- | --- | --- |
|  | Low expression No. (%) | High expression No. (%) |  |  |
| 1 | 7(87.5) | 1(12.5) | 8 | 0.137 |
| 2 | 15(100.0) | 0(0.0) | 15 |  |
| 3 | 68(83.0) | 14(17.0) | 82 |  |

**Table S2** Correlation between cytoplasmic REDD1 expression and tumor grade

in serous carcinomas

| Grade | Expression of REDD1 | | Total No | *P*-value |
| --- | --- | --- | --- | --- |
|  | Low expression No. (%) | High expression No. (%) |  |  |
| low | 4(40.0) | 6(60.0) | 10 | 0.751 |
| high | 54(47.0) | 61(53.0) | 115 |  |

| **Table S3** Nuclear REDD1 expression and OS | | | | | | | | | |  | | | | |  | |  |
| --- | --- | --- | --- | --- | --- | --- | --- | --- | --- | --- | --- | --- | --- | --- | --- | --- | --- |
| REDD1 expression | No. of patients | Median survival months | Survival rate (95% CI ) | | | | | | *χ^2^* | | | | *P*-value | | | |  |
|  |  | ( 95% CI ) | 36-months | | 60-months | | 120-months | |  | | | |  | | | |  |
| Negative | 186 | 107.700（84.829,130.571） | 0.67(0.592, 0.748) | | 0.48(0.383,0.578) | | 0.14(0.063,0.218) | | 1.238 | | | | 0.266 | | | |  |
| Positive | 43 | 86.300（37.503,135.097） | 0.55(0.374,0.725) | | 0.31(0.096,0.525) | | 0.18(0.054,0.414) | |  | | | |  | | | |  |
| **Table S4** Nuclear REDD1 expression and disease-free survival | | | | | | | | | | | |  | | | |  | |
| REDD1 expression | No. of patients | Median survival months | | Survival rate ( 95% CI ) | | | | | | | *χ^2^* | | | *P*-value | | | |
|  |  | ( 95% CI ) | | 36-months | | 60-months | | 120-months | | |  |  |  |  |  |  |  |
| Negative | 186 | 48.000(23.107，72.839) | | 0.51(0413, 0.608) | | 0.41(0.313,0.508) | | 0.08(0.022,0.139) | | | 0.059 | | | 0.808 | | | |
| Positive | 43 | 30.000(21.206，59.594) | | 0.34(0.126,0.555) | | 0.23(0.023,0.484) | | 0.23(0.023,0.484) | | |  | | |  | | | |
